# Maternal hypertensive disorders during pregnancy and the risk of offspring diabetes mellitus in childhood, adolescence, and early adulthood: a nationwide population-based cohort study

**DOI:** 10.1186/s12916-023-02762-5

**Published:** 2023-02-16

**Authors:** Liu Yang, Chen Huang, Min Zhao, Priscilla M. Y. Lee, Cheng Zhang, Yongfu Yu, Bo Xi, Jiong Li

**Affiliations:** 1 Department of Epidemiology, School of Public Health, Qilu Hospital, Cheeloo College of Medicine, Shandong University, Jinan, Shandong China; 2 Department of Biostatistics, School of Public Health, and The Key Laboratory of Public Health Safety of Ministry of Education, Fudan University, Shanghai, China; 3 Department of Nutrition and Food Hygiene, School of Public Health, Cheeloo College of Medicine, Shandong University, Jinan, China; 4 Department of Clinical Medicine - Department of Clinical Epidemiology, Aarhus University, Aarhus, Denmark; 5 Key Laboratory of Cardiovascular Remodeling and Function Research, Chinese Ministry of Education, Chinese National Health Commission and Chinese Academy of Medical Sciences, The State and Shandong Province Joint Key Laboratory of Translational Cardiovascular Medicine, Department of Cardiology, Qilu Hospital, Cheeloo College of Medicine, Shandong University, Jinan, China; 6 Shanghai Institute of Infectious Disease and Biosecurity, Shanghai, China; 7 Department of Epidemiology, School of Public Health, Nanjing Medical University, Nanjing, China

**Keywords:** Hypertension disorders during pregnancy, Diabetes, Offspring, Registers, Birth cohort

## Abstract

**Background:**

Maternal hypertensive disorders during pregnancy (HDP) have been suggested to contribute to the development of offspring cardiovascular disease later in life, but empirical evidence remains inconsistent. This study was aimed to assess the association of maternal overall and type-specific HDPs with diabetes in offspring from childhood to early adulthood.

**Methods:**

Using Danish national health registers, a total of 2,448,753 individuals born in Denmark from 1978 to 2018 were included in this study. Maternal HDP included chronic hypertension, gestational hypertension, and preeclampsia. The outcome of interest was diabetes in offspring (including type 1, type 2, and gestational diabetes). The follow-up of offspring started at birth and ended at the first diagnosis of diabetes, emigration from Denmark, death, or time end on 31 December 2018, whichever came first. Cox proportional hazards regression was used to evaluate the hazard ratios (HRs) with 95% confidence intervals (CIs) of the association between maternal HDP and diabetes (including type 1, type 2, and gestational diabetes) in offspring from birth to young adulthood (up to 41 years), with the offspring’s age as the time scale.

**Results:**

During a follow-up of up to 41 (median: 19.3) years, 1247 offspring born to mothers with HDP and 23,645 offspring born to mothers without HDP were diagnosed with diabetes. Compared with offspring born to mothers without HDP, those born to mothers with HDP had an increased risk for overall diabetes (HR=1.27, 95% CI=1.20–1.34), as well as for type 2 diabetes (HR=1.57, 95% CI=1.38–1.78) and gestational diabetes (HR=1.37, 95% CI=1.25–1.49). We did not observe obvious increased risk for type 1 diabetes (HR=1.08, 95% CI=0.98–1.18). Offspring of mothers with gestational hypertension (HR=1.37, 95% CI=1.00–1.88) or preeclampsia (HR=1.62, 95% CI=1.41–1.87) had higher risks of type 2 diabetes. The strongest association was observed for severe preeclampsia, with a 2-fold risk of type 2 diabetes (HR=2.00, 95% CI=1.42–2.82). The association between maternal HDP and type 1 diabetes did not reach statistical significance, except for maternal gestational hypertension (HR=1.41, 95%CI=1.17–1.71). In addition, we found that offspring born to mothers with any subtypes of maternal HDP had higher risk of gestational diabetes, and the corresponding HRs (95%CIs) for chronic hypertension, gestational hypertension, and preeclampsia were 1.60 (1.06–2.41), 1.29 (1.04–1.59), and 1.38 (1.24–1.53), respectively. We also observed stronger associations among offspring of mothers with HDP and comorbid diabetes (HR=4.64, 95%CI=3.85–5.60) than offspring of mothers with HDP or diabetes alone.

**Conclusions:**

Offspring of mothers with HDP, especially mothers with comorbid diabetes, had an increased risk of diabetes later in their life. Our findings suggest that timely and effective prevention of HDP in women of childbearing age should be taken into consideration as diabetes prevention and control strategies for their generations.

**Supplementary Information:**

The online version contains supplementary material available at 10.1186/s12916-023-02762-5.

## Background

Maternal hypertensive disorders during pregnancy (HDP), including chronic hypertension, gestational hypertension, and preeclampsia, are common complications during pregnancy [1]. HDP affects approximately 10% of pregnancies [2, 3] and remains one of the most critical issues in public health and perinatal medicine. Given the serious epidemic situation of HDP, it is urgent to determine any short- and long-term adverse effects of maternal HDP on the health of the offspring.

Substantial evidence has reported that women with HDP were at an increased long-term risk of cardiovascular disease, diabetes mellitus, and premature mortality [4–6]. Exposure to maternal HDP abnormal in utero environment may negatively impact the fetal functional expression of the hormonal axis and metabolites, increasing the risk of childhood obesity, endocrine disorders, and glucose metabolism abnormity, in offspring [7–9]. Although the association between specific maternal HDP and the development of diabetes in offspring has been reported [8, 10–14], most of the study has stemmed from case-control [12–14] or retrospective cohort studies [8], except for two prospective cohort with a relative small number of events (*n*<400) [10, 11]. Comprehensive studies assessing the effects of overall maternal HDP and its different subtypes on offspring diabetes within the same study population are still lacking. Regarding type-specific offspring diabetes, current evidence mainly focuses on type 2 diabetes [8, 10, 11]. Research on type 1 diabetes is limited to preeclampsia only [12–14]. Furthermore, evidence on the association of maternal HDP with offspring gestational diabetes remains scarce.

Our study aimed to comprehensively assess the effects of maternal overall HDP and its different subtypes (chronic hypertension, gestational hypertension, and preeclampsia) on diabetes mellitus and diabetes subtypes (including type 1, type 2, and gestational diabetes) in offspring from birth to young adulthood (age of up to 41 years) based on the Danish nationwide population-based cohort study. We further evaluated the combined effect of maternal HDP and maternal history of diabetes on the onset of offspring diabetes.

## Methods

### Ethics statement

This study was approved by the Data Protection Agency with Approval No. 2013-41-2569. According to Danish law, informed consent is not required for a register-based study based on de-identified data.

### Study population

The national health registries in Denmark provide the accurate linkage of personal information (including demographic data, socioeconomic factors, and medical records) to Danish residents by the assigned unique identification numbers [15]. The description in detail on various Danish registers has been described in Additional file 1: Table S1.

This population-based cohort study included all individuals born in Denmark between 1 Jan 1978 and 31 December 2018 (*N*=2,548,983). We excluded stillbirths (*n*=11,562), offspring with gestational age at birth <154 or >315 days (*n*=87,403), or offspring with missing information on sex (*n*=1265). A total of 2,448,753 mother-infant pairs were finally included for data analyses. The follow-up of offspring started at birth and ended if any of the following occurred: the first diagnosis of diabetes, emigration from Denmark, death, or time end on 31 December 2018, whichever came first.

### Exposures

Data on maternal HDP was extracted from the combination of the Danish National Patient Register and the Medical Birth Registry [15], using the International Classification of Diseases (ICD; ICD-8 during 1978–1993 and ICD-10 since 1994) (Additional file 1: Table S2). HDP was further categorized as moderate preeclampsia, severe preeclampsia, HELLP syndrome, unspecified preeclampsia, eclampsia, chronic hypertension, and gestational hypertension. Chronic hypertension [16] (ICD-8 codes 40009, 40019, 40029, 40039, 40099, 40199; ICD-10 codes I10-I13, I15, O10, O11) was defined as the diagnosis of hypertension before this pregnancy or before 20 weeks of gestation, and gestational hypertension [16] (ICD-8 codes 63700, 76029; ICD-10 codes O13, O16) as the diagnosis of hypertension after 20 weeks of gestation without proteinuria. The definition of preeclampsia has evolved since 2014 [17]. Of particular relevance to our study, as a sign of kidney involvement, proteinuria has no longer been required for a diagnosis of preeclampsia according to internationally accepted guidelines [17]. We extracted preeclampsia data from the combination of the Danish National Patient Register and the Medical Birth Registry [15] based on the ICD codes (ICD-8 codes 63703, 63704, 63709, 63719; ICD-10 codes O14.0, O14.1, O14.2, O14.9, O15). For women with more than one diagnosis of HDP subtypes, we categorized them based on the hierarchy: preeclampsia, chronic hypertension, and gestational hypertension [18]. It is worthy of note that, in this study, we only focused on HDP that occurred during the pregnancy for the index offspring, which means that, for each offspring, if the mother was diagnosed with HDP during the corresponding pregnancy, then we defined this offspring to be exposed, and if the mother was not diagnosed with HDP during the corresponding pregnancy (including the mother being diagnosed with HDP in other pregnancies), then we defined this offspring to be unexposed.

### Outcomes

The outcome of interest was diabetes in offspring, including type 1 diabetes, type 2 diabetes, and gestational diabetes. Information on diabetes was retrieved from the Danish National Diabetes Register, the Danish National Patient Registry, and the Danish National Prescription Registry [15], using the ICD codes and Anatomical Therapeutic Chemical (ATC) classification codes (ICD-8 249, 250, 63.74, Y6449; ICD-10 E10, E11, O24, H36.0; ATC A10A, A10B) (Additional file 1: Table S3). Given that diabetes was recorded using a single code (250) through 1978–1986, two approaches were used to distinguish type 1 and type 2 diabetes during this period: (1) the specific code for type 1 or type 2 diabetes applied later or (2) the age of onset of diabetes (onset <30 years for type 1, and ≥30 years for type 2) [19, 20].

### Covariates

Potential confounders included calendar year of birth (1978–1987, 1988–1997, 1998–2007, and 2008–2018), maternal age at delivery (<20, 20–24, 25–29, 30–34, and ≥35 years), maternal country of origin (Danish origin vs. other), maternal residence at birth (Copenhagen, big cities with ≥100,000 inhabitants, and other), maternal educational level (0–9, 10–14, and ≥15 years), maternal cohabitation at birth (single vs. cohabitating), maternal income categories at birth (no income, three tertiles), maternal pre-pregnancy BMI (<18.5 kg/m^2^ [underweight], 18.5–24.9 kg/m^2^ [normal weight], 25.0–29.9 kg/m^2^ [overweight], ≥30.0 kg/m^2^ [obesity]), maternal smoking (yes vs. no), singleton status (yes vs. no), parity (1, 2, and ≥3 children), sex of offspring (male vs. female), maternal diabetes history before childbirth (yes vs. no), and paternal diabetes history before childbirth (yes vs. no). Data on maternal and birth characteristics were extracted from the Danish Medical Birth Register [15], and socioeconomic factors were extracted from the Danish Integrated Database for Longitudinal Labour Market Research and the Danish Civil Registry System [15]. Data on parental history of disease were extracted from the Danish National Patient Registry [15]. Missing data on covariates were treated with multiple imputation with five replicates.

### Statistical analysis

Considering non-diabetes deaths as competing events, we performed competing risk analyses to estimate the adjusted cumulative incidence of diabetes among offspring exposed and unexposed to maternal HDP using the inverse probability of treatment weighting approach. An evaluation of proportional hazard assumption using log-minus-log plots suggested there was no obvious violation. Cox proportional hazards regression models were used to calculate the HRs with 95% CIs to assess the association between maternal HDP and diabetes in offspring, with the offspring’s age as time scale. For type-specific diabetes, we modeled the different subtypes of diabetes individually and censored those participants diagnosed with other types of diabetes at the date of diagnosis. When focusing on gestational diabetes in the offspring, we restricted the analysis to female offspring who had been pregnant and date of follow-up started at the age of 15 years old [21]. Interaction terms of maternal HDP with maternal diabetes history on diabetes in offspring were tested to assess whether the association was varied by a history of maternal diabetes. We assessed the association according to the timing of maternal HDP diagnosis (diagnosed before childbirth and diagnosed ≤ 1 year, 2 to 3 years, 4 to 10 years, and 11 to 15 years after childbirth) compared with no maternal HDP. We split each observation into segments of time intervals according to the age period of offspring (0–9, 10–14, 15–19, 20–24, and ≥25 years) and examined the association between maternal HDP and diabetes in offspring stratified by the age group of the offspring [22].

Furthermore, we sequentially performed the following five sensitivity analyses to test the robustness of our findings: (1) to assess the influence of potential confounders mentioned above on the association between maternal HDP and diabetes in offspring, we performed stratified analyses. (2) We additionally adjusted for paternal hypertension history. (3) We repeated the main analysis restricting the study population to offspring born after 1991 with adjustment for maternal smoking (data on maternal smoking was available since 1991). (4) Given the possibility of misdiagnosis, we performed analysis after excluding mothers diagnosed with multiple types of hypertension in one pregnancy. (5) To explore the influence of maternal obesity, we adjusted for maternal obesity before childbirth and repeated the analysis. (6) We restricted analysis to offspring born after 1994 to evaluate the influence of the cover periods of different registers. (7) We have additionally adjusted for low birth weight to explore the potential influence of birth weight on the association. (8) Furthermore, given that the preeclampsia concept has evolved since 2014, we have restricted analysis to offspring born before 2014 to evaluate the impact of redefined preeclampsia concept. (9) Considering the potential misclassification between type 1 and type 2 diabetes using age onset of diabetes as an additional definition when the specific code was not available, we re-conducted the main analysis excluding those diabetic patients identified through age. All analyses were performed using SAS 9.4 (SAS Institute, Cary, NC) and Stata 15.1 (StataCorp, College Station, TX, USA). A two-tailed *P* value <0.05 was considered statistically significant.

## Results

Among all 2,448,753 individuals born during 1978 to 2018, 102,698 (4.19%) were exposed to mothers with HDP (chronic hypertension: 0.63%, gestational hypertension: 0.76%, and preeclampsia: 2.81%, respectively). A total of 90,165 offspring (3.68%) were censored through follow-up, of which 66,818 (2.73%) were owing to emigration and 23,347 (0.95%) were owing to non-diabetes deaths. Compared with mothers without HDP, those with HDP were more likely to be older, primiparous, and overweight or obese, to smoke less during pregnancy, and to have a history of diabetes, and no singleton birth. Offspring exposed to maternal HDP tended to have a higher proportion of paternal history of diabetes and hypertension (Table 1).

**Table 1:** Baseline characteristics of the study participants according to maternal HDP

Characteristics	No HDP	Preeclampsia or eclampsia	Chronic hypertension	Gestational hypertension	Total
***N***	2,346,055 (95.8)	68,800 (2.8)	15,336 (0.6)	18,562 (0.8)	2,448,753 (100.0)
**Outcome**					
No	2,322,410 (99.0)	67,881 (98.7)	15,246 (99.4)	18,324 (98.7)	2,423,861 (99.0)
Yes	23,645 (1.0)	919 (1.3)	90 (0.6)	238 (1.3)	24,892 (1.0)
**Singleton**					
No	74,687 (3.2)	6350 (9.2)	677 (4.4)	811 (4.4)	82,525 (3.4)
Yes	2,271,368 (96.8)	62,450 (90.8)	14,659 (95.6)	17,751 (95.6)	2,366,228 (96.6)
**Sex**					
Male	1,203,772 (51.3)	35,799 (52.0)	7908 (51.6)	9638 (51.9)	1,257,117 (51.3)
Female	1,142,283 (48.7)	33,001 (48.0)	7428 (48.4)	8924 (48.1)	1,191,636 (48.7)
**Calendar year of birth**					
1978–1987	478,305 (20.4)	14,923 (21.7)	709 (4.6)	3569 (19.2)	497,506 (20.3)
1988–1997	625,820 (26.7)	18,144 (26.4)	1551 (10.1)	3589 (19.3)	649,104 (26.5)
1998–2007	618,242 (26.4)	16,390 (23.8)	4981 (32.5)	4258 (22.9)	643,871 (26.3)
2008–2018	623,688 (26.6)	19,343 (28.1)	8095 (52.8)	7146 (38.5)	658,272 (26.9)
**Maternal country of origin**					
Others	284,848 (12.1)	5477 (8.0)	1337 (8.7)	1317 (7.1)	292,979 (12.0)
Danish origin	2,057,394 (87.7)	63,236 (91.9)	13,992 (91.2)	17,217 (92.8)	2,151,839 (87.9)
Unknown	3813 (0.2)	87 (0.1)	7 (0.1)	28 (0.2)	3935 (0.2)
**Maternal parity**					
1	1,045,191 (44.6)	45,295 (65.8)	5094 (33.2)	10,939 (58.9)	1,106,519 (45.2)
2	879,045 (37.5)	15,920 (23.1)	6477 (42.2)	4974 (26.8)	906,416 (37.0)
≥3	421,819 (18.0)	7585 (11.0)	3765 (24.6)	2649 (14.3)	435,818 (17.8)
**Maternal age at childbirth (years)**					
<20	51,693 (2.2)	1990 (2.9)	46 (0.3)	253 (1.4)	53,982 (2.2)
20–24	398,027 (17.0)	13,852 (20.1)	953 (6.2)	2637 (14.2)	415,469 (17.0)
25–29	853,251 (36.4)	24,604 (35.8)	3731 (24.3)	6148 (33.1)	887,734 (36.3)
30–34	715,587 (30.5)	18,162 (26.4)	5698 (37.2)	5692 (30.7)	745,139 (30.4)
35+	327,497 (14.0)	10,192 (14.8)	4908 (32.0)	3832 (20.6)	346,429 (14.1)
**Maternal BMI during pregnancy**					
<18.5	37,158 (4.3)	578 (2.2)	239 (2.3)	160 (1.8)	38,135 (4.2)
18.5–24.9	524,766 (60.4)	11,694 (45.4)	4699 (44.5)	3754 (41.5)	544,913 (59.6)
25.0–29.9	175,210 (20.2)	6554 (25.4)	2495 (23.6)	2316 (25.6)	186,575 (20.4)
≥30.0	99,285 (11.4)	6181 (24.0)	2793 (26.4)	2593 (28.7)	110,852 (12.1)
Unknown	32,208 (3.7)	775 (3.0)	343 (3.3)	219 (2.4)	33,545 (3.7)
**Maternal smoking during pregnancy** ^a^					
No	1,319,267 (78.0)	39,592 (81.2)	12,040 (84.1)	11,751 (84.4)	1,382,650 (78.2)
Yes	314,002 (18.6)	6863 (14.1)	1768 (12.4)	1739 (12.5)	324,372 (18.3)
Unknown	58,196 (3.4)	2288 (4.7)	505 (3.5)	433 (3.1)	61,422 (3.5)
**Maternal education at childbirth, years**					
0–9	600,423 (25.6)	19,001 (27.6)	2669 (17.4)	4059 (21.9)	626,152 (25.6)
10–14	1,004,808 (42.8)	30,677 (44.6)	6759 (44.1)	8342 (44.9)	1,050,586 (42.9)
15+	701,709 (29.9)	18,328 (26.6)	5760 (37.6)	5919 (31.9)	731,716 (29.9)
Unknown	39,115 (1.7)	794 (1.2)	148 (1.0)	242 (1.3)	40,299 (1.6)
**Maternal cohabitation at childbirth**					
No	1,074,662 (45.8)	35,675 (51.9)	6861 (44.7)	9024 (48.6)	1,126,222 (46.0)
Yes	1,270,421 (54.2)	33,102 (48.1)	8473 (55.3)	9530 (51.3)	1,321,526 (54.0)
Unknown	972 (0.04)	23 (0.03)	2 (0.01)	8 (0.04)	1005 (0.00)
**Maternal residence at birth**					
Copenhagen	274,998 (11.7)	7778 (11.3)	1577 (10.3)	2121 (11.4)	286,474 (11.7)
Big cities ≥ 100,000 inhabitants	307,175 (13.1)	9446 (13.7)	2014 (13.1)	3034 (16.4)	321,669 (13.1)
Other	1,763,882 (75.2)	51,576 (75.0)	11,745 (76.6)	13,407 (72.2)	1,840,610 (75.2)
**Maternal income at birth**					
Less than lowest tertiles	424,799 (18.8)	10,599 (15.9)	2530 (16.6)	2524 (14.1)	440,452 (18.6)
Lowest and middle tertiles	611,191 (27.0)	19,029 (28.6)	3495 (22.9)	4616 (25.7)	638,331 (27.0)
Middle and highest tertiles	611,914 (27.1)	18,913 (28.4)	4596 (30.1)	5489 (30.6)	640,912 (27.1)
More than highest tertiles	613,931 (27.1)	17,944 (27.0)	4649 (30.4)	5301 (29.6)	641,825 (27.2)
Unknown	515 (0.02)	9 (0.01)	1 (0.01)	3 (0.02)	528 (0.02)
**Paternal diabetes history before childbirth**					
No	2,309,753 (98.5)	67,379 (97.9)	15,004 (97.8)	18,177 (97.9)	2,410,313 (98.4)
Yes	13,993 (0.6)	494 (0.7)	141 (0.9)	155 (0.8)	14,783 (0.6)
Unknown	22,309 (1.0)	927 (1.4)	191 (1.3)	230 (1.2)	23,657 (1.0)
**Maternal diabetes history before childbirth**					
No	2,306,898 (98.3)	65,825 (95.7)	13,932 (90.9)	17,539 (94.5)	2,404,194 (98.2)
Yes	39,157 (1.7)	2975 (4.3)	1404 (9.2)	1023 (5.5)	44,559 (1.8)
**Paternal hypertension history before childbirth**					
No	2,313,170 (98.6)	67,514 (98.1)	15,011 (97.9)	18,216 (98.1)	2,413,911 (98.6)
Yes	10,576 (0.5)	359 (0.5)	134 (0.9)	116 (0.6)	11,185 (0.5)
Unknown	22,309 (1.0)	927 (1.4)	191 (1.3)	230 (1.2)	23,657 (1.0)

^a^Information on maternal smoking during pregnancy was available from 1991 to 2018

**Fig. 1:**
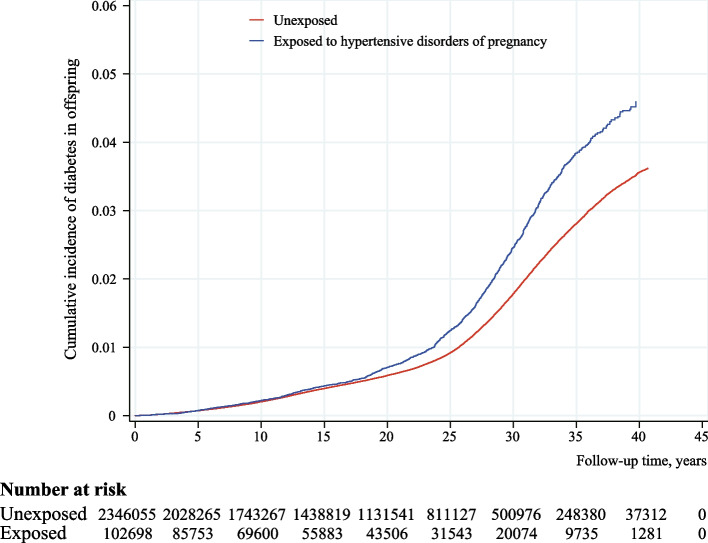
Adjusted cumulative incidence of diabetes among offspring exposed vs. unexposed to maternal HDP. *Note:* Adjusted cumulative incidence was averaged across the distribution of the covariates (calendar year, maternal age, maternal country of origin, maternal residence at birth, maternal cohabitation at birth, maternal educational level, maternal income categories at birth, maternal pre-pregnancy BMI, maternal smoking status during pregnancy, singleton status, maternal diabetes history before childbirth, paternal diabetes history before childbirth, parity, and sex of offspring) using the inverse probability of treatment weighting approach

During the follow-up period of up to 41 years (median: 19.3 years, IQR: 9.6 to 28.5 years), 1247 offspring born to mothers with HDP and 23,645 offspring born to mothers without HDP were diagnosed with diabetes. Adjusted cumulative incidence of offspring diabetes among offspring born to mothers with or without HDP at 40 years of age were 4.59% (4.25 to 4.95%) and 3.62% (3.55 to 3.69%), respectively (Fig. 1). Compared with offspring unexposed to maternal HDP, those exposed to maternal HDP had a 27% higher risk of developing diabetes in their first 41 years of life (HR=1.27, 95%CI=1.20–1.34). We observed increased risks of type 2 diabetes (HR=1.57, 95%CI=1.38–1.78) and gestational diabetes (HR=1.37, 95%CI=1.25–1.49) for offspring exposed to maternal HDP, while there was no obvious increased risk for type 1 diabetes (HR=1.08, 95%CI=0.98–1.18). The risk of type 2 diabetes was higher among offspring exposed to gestational hypertension (HR=1.37, 95%CI=1.00–1.88) and preeclampsia (HR=1.62, 95%CI=1.41–1.87), particularly for severe preeclampsia (HR=2.00, 95%CI=1.42–2.82). We only found offspring born to mothers with gestational hypertension had a higher risk of type 1 diabetes (HR=1.41, 95%CI=1.17–1.71), compared with mothers without maternal HDP. In addition, the risks for gestational diabetes were also increased among offspring exposed to any type-specific HDP, with higher HRs seen in chronic hypertension (HR=1.60, 95%CI=1.06–2.41), gestational hypertension (HR=1.29, 95%CI=1.04–1.59), and preeclampsia (HR=1.38, 95%CI=1.24–1.53), respectively (Table 2). Besides, an increased risk of developing diabetes was seen in offspring born to mothers who had HDP and comorbid diabetes (HR=4.64, 95%CI=3.85–5.60), compared with those born to mothers with either only, or with neither HDP nor diabetes history (Table 3).

**Table 2:** Association between maternal hypertensive disorders and diabetes in offspring

Outcomes of offspring	Exposure	No. of diabetes cases	Rate (1/1000 person-years)	cHR^**a**^ (95%CI)	aHR^**b**^ (95%CI)
**Diabetes mellitus**	**No maternal HDP**	23,645	0.52	1.00 (Reference)	1.00 (Reference)
	**Maternal HDP**	1247	0.68	1.33 (1.26–1.41)	1.27 (1.20–1.34)
	**Preeclampsia or eclampsia**	919	0.69	1.30 (1.22–1.39)	1.25 (1.17–1.33)
	**Preeclampsia**	915	0.69	1.31 (1.22–1.40)	1.25 (1.17–1.34)
	Moderate	713	0.71	1.29 (1.20–1.39)	1.25 (1.16–1.34)
	Severe	135	0.65	1.38 (1.16–1.63)	1.28 (1.08–1.51)
	HELLP syndrome^*^	–	–	–	–
	Unspecified	63	0.75	1.41 (1.10–1.80)	1.34 (1.05–1.72)
	Eclampsia^*^	–	–	–	–
	**Hypertension**	328	0.66	1.43 (1.29–1.60)	1.33 (1.19–1.48)
	Chronic	90	0.50	1.44 (1.17–1.78)	1.22 (0.99–1.51)
	Gestational	238	0.75	1.43 (1.26–1.63)	1.37 (1.21–1.56)
**Type 1 diabetes**	**No maternal HDP**	10,599	0.23	1.00 (Reference)	1.00 (Reference)
	**Maternal HDP**	493	0.27	1.16 (1.06–1.27)	1.08 (0.98–1.18)
	**Preeclampsia or eclampsia**	325	0.24	1.05 (0.94–1.17)	0.99 (0.89–1.11)
	**Preeclampsia**	325	0.25	1.06 (0.95–1.18)	1.00 (0.90–1.12)
	Moderate	252	0.25	1.07 (0.94–1.21)	1.02 (0.90–1.16)
	Severe	50	0.24	1.03 (0.78–1.36)	0.95 (0.72–1.26)
	Unspecified	19	0.23	0.97 (0.62–1.52)	0.95 (0.60–1.49)
	**Hypertension**	168	0.34	1.48 (1.27–1.73)	1.28 (1.10–1.50)
	Chronic	56	0.31	1.39 (1.07–1.81)	1.08 (0.83–1.41)
	Gestational	112	0.35	1.53 (1.27–1.85)	1.41 (1.17–1.71)
**Type 2 diabetes**	**No maternal HDP**	3872	0.09	1.00 (Reference)	1.00 (Reference)
	**Maternal HDP**	252	0.14	1.66 (1.46–1.89)	1.57 (1.38–1.78)
	**Preeclampsia or eclampsia**	202	0.15	1.74 (1.51–2.00)	1.62 (1.41–1.87)
	**Preeclampsia**	200	0.15	1.73 (1.50–2.00)	1.62 (1.41–1.87)
	Moderate	156	0.15	1.70 (1.45–1.99)	1.58 (1.35–1.86)
	Severe	33	0.16	2.13 (1.51–3.00)	2.00 (1.42–2.82)
	Unspecified	11	0.13	1.48 (0.82–2.67)	1.39 (0.77–2.51)
	**Hypertension**	50	0.10	1.41 (1.07–1.87)	1.37 (1.03–1.81)
	Chronic	11	0.06	1.35 (0.75–2.45)	1.36 (0.75–2.46)
	Gestational	39	0.12	1.43 (1.04–1.96)	1.37 (1.00–1.88)
**Gestational diabetes**^†^	**No maternal HDP**	9174	1.14	1.00 (Reference)	1.00 (Reference)
	**Maternal HDP**	502	1.62	1.42 (1.30–1.55)	1.37 (1.25–1.49)
	**Preeclampsia or eclampsia**	392	1.62	1.42 (1.29–1.57)	1.37 (1.24–1.52)
	**Preeclampsia**	390	1.62	1.43 (1.29–1.58)	1.38 (1.24–1.53)
	Moderate	305	1.58	1.38 (1.23–1.55)	1.34 (1.19–1.50)
	Severe	52	1.70	1.54 (1.18–2.03)	1.47 (1.12–1.94)
	Unspecified	33	1.97	1.82 (1.30–2.57)	1.65 (1.17–2.32)
	**Hypertension**	110	1.61	1.39 (1.16–1.68)	1.34 (1.11–1.62)
	Chronic	23	1.94	1.80 (1.19–2.71)	1.60 (1.06–2.41)
	Gestational	87	1.54	1.32 (1.07–1.63)	1.29 (1.04–1.59)

*aHR* adjusted hazard ratio, *cHR* crude hazard ratio, *HDP* hypertensive disorders during pregnancy, *HELLP* hemolysis, elevated liver enzymes, and low platelet, *CI* confidence interval

^a^Model 1: using offspring’s age as time scale

^b^Model 2: using offspring’s age as time scale; adjusted for calendar year of birth, maternal age at birth, maternal country of origin, maternal residence at birth, maternal cohabitation at birth, maternal educational level, maternal income categories at birth, maternal pre-pregnancy BMI, maternal smoking status during pregnancy, singleton status, maternal diabetes history before childbirth, paternal diabetes history before childbirth, parity, and sex of offspring

*Number of cases for HELLP syndrome and eclampsia are less than 6 and not allowed to report due to privacy protection; therefore, we did not report results of HELLP syndrome and eclampsia

^†^The analysis of gestational diabetes was restricted to female offspring who had been pregnant

**Table 3:** Association of interaction between maternal hypertensive disorders and maternal diabetes history with diabetes in offspring

Outcomes	Maternal HDP	Maternal diabetes	No. of diabetes cases	Rate (1/1000 person-years)	cHR^**a**^ (95%CI)	aHR^**b**^ (95%CI)
**Diabetes mellitus**	No	No	23,004	0.51	1.00 (Reference)	1.00 (Reference)
	Yes	No	1137	0.64	1.27 (1.20–1.35)	1.27 (1.19–1.35)
	No	Yes	641	1.43	3.87 (3.57–4.18)	3.67 (3.39–3.97)
	Yes	Yes	110	1.79	4.85 (4.02–5.85)	4.64 (3.85–5.60)
**Type 1 diabetes**	No	No	10,267	0.23	1.00 (Reference)	1.00 (Reference)
	Yes	No	433	0.24	1.08 (0.98–1.19)	1.05 (0.96–1.16)
	No	Yes	332	0.74	3.44 (3.08–3.83)	3.12 (2.79–3.48)
	Yes	Yes	60	0.97	4.55 (3.53–5.86)	4.04 (3.13–5.22)
**Type 2 diabetes**	No	No	3718	0.08	1.00 (Reference)	1.00 (Reference)
	Yes	No	230	0.13	1.60 (1.40–1.83)	1.63 (1.43–1.87)
	No	Yes	154	0.34	6.68 (5.68–7.85)	6.99 (5.94–8.22)
	Yes	Yes	22	0.36	6.95 (4.57–10.57)	7.41 (4.87–11.27)
**Gestational diabetes**^†^	No	No	9019	1.13	1.00 (Reference)	1.00 (Reference)
	Yes	No	474	1.56	1.38 (1.26–1.51)	1.36 (1.24–1.50)
	No	Yes	155	3.74	3.69 (3.15–4.33)	3.35 (2.85–3.95)
	Yes	Yes	28	4.94	5.07 (3.50–7.34)	4.67 (3.22–6.77)

*aHR* adjusted hazard ratio, *cHR* crude hazard ratio, *HDP* hypertensive disorders during pregnancy, *CI* confidence interval

^a^Model 1: using offspring’s age as time scale

^b^Model 2: using offspring’s age as time scale; adjusted for calendar year of birth, maternal age, maternal country of origin, maternal residence at birth, maternal cohabitation at birth, maternal educational level, maternal income categories at birth, maternal pre-pregnancy BMI, maternal smoking status during pregnancy, singleton status, paternal diabetes history before childbirth, parity, and sex of offspring

^†^The analysis of gestational diabetes was restricted to female offspring who had been pregnant

**Fig. 2:**
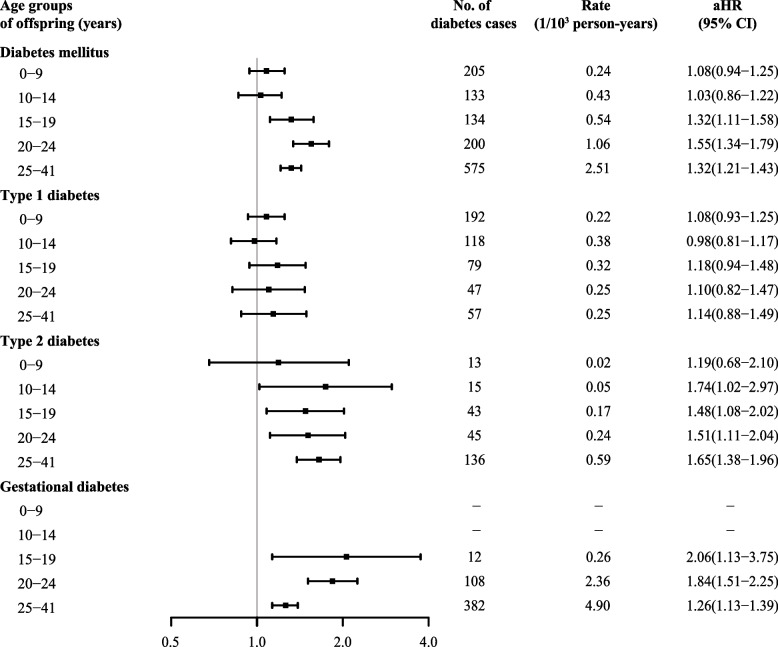
Associations between maternal HDP and diabetes in offspring by offspring’s age and HDP subtype. *Note:* The analysis of gestational diabetes was restricted to female offspring who had been pregnant; Adjusted for calendar year of birth, maternal age, maternal country of origin, maternal residence at birth, maternal cohabitation at birth, maternal educational level, maternal income categories at birth, maternal pre-pregnancy BMI, maternal smoking status during pregnancy, singleton status, maternal diabetes history before childbirth, paternal diabetes history before childbirth, parity, and sex of offspring

**Fig. 3:**
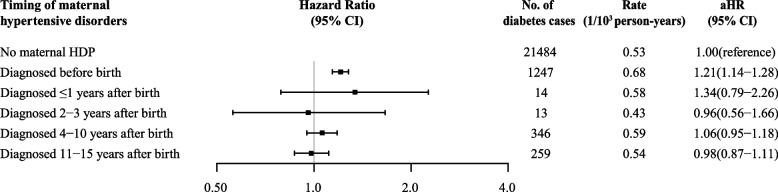
Associations between maternal HDP of pregnancy and diabetes in offspring, according to the timing of the maternal HDP diagnosis. *Note:* Adjusted for calendar year of birth, maternal age, maternal country of origin, maternal residence at birth, maternal cohabitation at birth, maternal educational level, maternal income categories at birth, maternal pre-pregnancy BMI, maternal smoking status during pregnancy, singleton status, maternal diabetes history before childbirth, paternal diabetes history before childbirth, parity, and sex of offspring

An increased risk of type 2 diabetes was observed for offspring exposed to maternal HDP in early adolescence (10 to 14 years: HR=1.74, 95%CI=1.02–2.97), late adolescence (15–19 years: HR=1.48, 95%CI=1.08–2.02), and early adulthood (20–24 years: HR=1.51, 95%CI=1.11–2.04, 25 to 41 years: HR=1.65, 95%CI=1.38–1.96) (Fig. 2). We also observed an increased risk of gestational diabetes for offspring exposed to maternal HDP and the association was attenuated (but still significant) with the increased age (15–19 years: HR=2.06, 95%CI=1.13–3.75; 20–24 years: HR=1.84, 95%CI=1.51–2.25; and 25 to 41 years: HR=1.26, 95%CI=1.13–1.39) (Fig. 2). In terms of diagnostic time of maternal HDP, compared with mothers without HDP, an increased risk was found only when maternal HDP was diagnosed before birth (HR=1.21, 95%CI=1.14–1.28) (Fig. 3).

Subgroup analyses showed that the positive association between maternal HDP and offspring diabetes was largely similar in each subgroup by baseline characteristics (Additional file 1: Table S4). Sensitivity analyses restricted to offspring born after 1991 for maternal smoking adjustment, excluded offspring of mothers who diagnosed multiple types of HDP in one pregnancy, additionally adjusted for paternal hypertension, or adjusted for maternal obesity before childbirth, restricted analysis to offspring born after 1994 for the capture of diabetes diagnoses in same period in different registers, to offspring born before 2014 for the redefined preeclampsia concept, additionally adjusting for low birth weight, and excluded those diabetic patients identified through age from the main analysis, yielded similar results to the primary analyses (Additional file 1: Tables S5-9).

## Discussion

Using this large population-based cohort study, we found that offspring exposure to maternal HDP had higher risk of diabetes, and was associated with 56% and 37% increased risks of offspring type 2 diabetes and gestational diabetes, respectively. However, we uncovered no association between maternal HDP and type 1 diabetes in offspring. The occurrence of maternal HDP, either gestational hypertension or preeclampsia, was associated with an increased risk of offspring type 2 diabetes. An increased risk of type 1 diabetes was solely observed in offspring exposed to gestational hypertension. In addition, offspring exposed to any subtypes of maternal HDP would have increased risks of gestational diabetes. We also observed stronger associations among offspring born to mothers with HDP and comorbid diabetes.

To our knowledge, limited studies comprehensively evaluated the effect of maternal HDP on offspring diabetes [8, 23]. A meta-analysis including 11,050,451 offspring of 13 studies reported that preeclampsia was associated with a weak increased risk of offspring diabetes (rate ratio=1.12, 95%CI=0.98–1.27) [23]. However, the significant heterogeneity between studies (*I*^*2*^: 48.2%, *P*=0.02) limited the reliability of results. Another population-based retrospective study of 232,841 singleton deliveries between 1991 to 2014 in Israel showed that compared with young offspring (≤18 years) born to normotensive women, offspring born to women with chronic hypertension had 1.39-fold increased risk of diabetes with a relative wide confidence interval (95%CI=0.52–3.73) due to a small number of events (*n*=4) [8]. In addition, the abovementioned studies only assessed the effect of maternal type-specific HDP on offspring overall diabetes. In our study, we comprehensively assessed the association of maternal HDP and its subtypes (preeclampsia, chronic hypertension, and gestational hypertension) with diabetes in offspring from birth to young adulthood (up to 41 years old) and found an increased risk of diabetes in offspring born to mothers with overall or type-specific HDP.

We also found that the offspring of mothers with either gestational hypertension or preeclampsia had an increased risk of offspring type 2 diabetes. Consistent with our findings, a study composed of 5335 older adults born in 1934–1944 from the Helsinki Birth Cohort Study reported that offspring exposed to maternal gestational hypertension in utero had a 1.14-fold risk of type 2 diabetes at 50–61 years [10]. But this study did not report obvious risk for type 2 diabetes among offspring of mothers with severe (HR=1.02, 95%CI=0.69–1.53) or non-severe preeclampsia (HR=0.94, 95%CI=0.58–1.53) [10]. However, a previous cohort study including 8648 offspring born between 1952 and 1958 based on the Walker Cohort reported an adverse effect of maternal preeclampsia on type 2 diabetes in offspring (odds ratio=1.38, 95%CI=0.89–2.13) [11]. The inconsistent findings abovementioned might be attributed to the baseline characteristics of the study populations, duration of follow-up, statistical power, adjusted covariates, and identification of diabetes. In the present study, based on data from several national registers of Denmark, we ascertained and verified diabetes with high reliability [24]. After comprehensively adjusting for several maternal and birth characteristics and socioeconomic factors, we assessed the effects of overall and type-specific maternal HDP on offspring type 2 diabetes using the large prospective cohort study (*n*=2,448,753). Type 2 diabetes, usually refers to as adult-onset disease, is partially determined by the accumulation of risk factors during early development [25]. We observed positive associations between maternal HDP and type 2 diabetes through adolescence and early adulthood (≥10 years), and this adverse effect seemed to enhance with increased age.

Earlier studies in 1990s have reported that preeclampsia was associated with offspring type 1 diabetes [12, 13]. However, a case-control designed register study including 14,949 type 1 diabetes cases onset at ages 0–14 years from the Swedish Childhood Diabetes Register and 55,712 matched controls from the Swedish Total Population Register born from 1973 to 2013 [14], as well as the present population-based register study, covered a contemporary considerable time span of birth cohorts, showed no association of maternal preeclampsia with offspring type 1 diabetes. The inconsistent findings might be explained by new and more active pregnancy treatments, which might eliminate some of the adverse effects of preeclampsia. Simultaneously, we observed obvious increased risk for type 1 diabetes in the offspring of mothers with gestational hypertension for the first time. The differences in the association between maternal HDP and type-specific diabetes in offspring may be owing to the effects of various future risk factors and differential pathogenesis for type 1 (organ-specific immune destruction of the pancreatic β-islets) [26] and type 2 diabetes (insulin resistance) [27]. Further studies on the underlying mechanisms and other potential risk factors throughout life for offspring type 1 diabetes are warranted.

We also observed an increased risk of gestational diabetes among offspring exposed to maternal overall and any subtypes of HDP, compared with those who were not exposed to maternal HDP. Evidence suggested that women with high blood pressure before or during pregnancy tended to have an increased risk of gestational diabetes [28], and several common pathogenic pathways might be underlying the association including insulin resistance [29, 30], endothelial dysfunction [31, 32], and inflammation markers [33, 34]. Offspring exposed to higher levels of glucose spectrum in utero are more likely to be insulin resistant and limited β-cell compensation [35, 36]. Such modulation in girls might increase the propensity to develop gestational diabetes.

The underlying mechanisms between maternal HDP and diabetes in offspring are not fully elucidated. Maternal HDP has been suggested to be associated with non-specific systemic inflammatory reaction and circulating cortisol levels overexposure, which further result in hypoxia and fetal malnutrition [37, 38]. Evidence showed that perturbed maternal-fetal environment was associated with the development of adult diabetes according to the modulation of epigenetic regulation of gene expression [25]. Methylation presence has been identified among women with preeclampsia [39], and in cord blood [40], which could decrease the numbers of stem cells involved in islet cell development, with an increased risk for abnormal insulin secretion, resistance to insulin, and type 2 diabetes in offspring [39]. Thus, epigenetic changes might have an essential role in the association between maternal HDP and diabetes in offspring.

We found that mothers with both HDP and diabetes history tended to have a higher risk of offspring diabetes, compared with offspring born to mothers without HDP and diabetes history. Further research on the added effects of coexisting maternal HDP and maternal diabetes history on offspring diabetes is required to evaluate the burden of multimorbidity through pregnancy.

## Strengths and limitations

This study has several strengths. First, we used the large population-based cohort study based on the Danish national registries, which covered all Danish residents with a long follow-up duration of up to 41 years, thus providing sufficient power and minimizing the possibility of selection and recall bias to obtain reliable statistical estimates. Second, we comprehensively assessed the association of maternal HDP and its different subtypes on diabetes in offspring from birth to young adulthood. Third, the availability of maternal and birth baseline characteristics and socioeconomic factors from the several Danish registration systems enables us to adjust for a wide range of important covariables. However, several limitations should be noted. First, although we have adjusted for a large number of important confounding factors, residual confounding caused by unmeasured confounders in childhood or adulthood, such as smoking status, alcohol intake, physical activity, sleep quality, and body mass index, may influence our findings. However, further adjustment for paternal hypertension in sensitivity analyses yielded similar results as the primary analyses, which suggested that the observed associations are unlikely to be completely attributable to unmeasured confounders. Second, there might be potential misclassification in the diagnosis of maternal HDP and offspring diabetes. In addition, due to the periods captured in the different registries and unavailable specific codes for type 1 and type 2 diabetes among offspring born from 1978 to 1986, potential misclassification might exist for subtypes of diabetes. However, we performed sensitivity analyses restricted to offspring born after 1994, excluded those diabetic patients identified through age from the main analysis, respectively, and found similar results to the primary analyses. Previous validation studies showed high reliability for ascertainment of maternal HDP (sensitivity: 69%, specificity: 99%) [18, 41] and offspring diabetes (sensitivity ≥95%) in Denmark [24]. Third, as our study was conducted in Denmark, the generality of our findings to other countries should be made cautiously. Fourth, the presentation of the mediation analysis of birthweight might have a potential limitation, as it is prone to residual confounding of the mediator/outcome relationship and collider bias.

## Conclusions

In conclusion, our study provides evidence that offspring born to mothers with HDP, especially mothers with comorbid diabetes, had higher risk of diabetes later in life, especially for type 2 diabetes and gestational diabetes. Maternal HDP did not increase the risk of offspring type 1 diabetes, except for gestational hypertension. These findings emphasize the importance and necessity of screening HDP status in women of childbearing age to identify and control maternal HDP early, thereby reducing the risk of diabetes in their offspring.

## Supplementary Information

**Additional file 1: Table S1.** The information of registers used in this study. **Table S2.** Detailed information of methods used to identify maternal hypertensive disorders during pregnancy. **Table S3.** Detailed information of methods used to identify offspring diabetes. **Table S4.** Associations between maternal HDP and diabetes in offspring by baseline characteristics. **Table S5.** Sensitivity analyses of the association between maternal HDP and diabetes in offspring. **Table S6.** Association between maternal HDP and diabetes in offspring born after 1994. **Table S7.** Associations between maternal HDP and diabetes in offspring with additional adjustment for low birth weight. **Table S8.** Association between maternal HDP and diabetes in offspring born before 2014. **Table S9.** Associations between maternal HDP and diabetes in offspring after excluding those diabetic patients identified through age.

## Data Availability

Data collected for this study and additional related documents will be available to others by contacting the corresponding author (Prof. Yongfu Yu, Email: yu@fudan.edu.cn).

## Abbreviations

ATC: Anatomical Therapeutic Chemical
CI: Confidence interval
HR: Hazard ratio
HDP: Hypertensive disorders during pregnancy
ICD: International Classification of Diseases
