# Supplementary material for: Maternal hypertensive disorders during pregnancy and the risk of offspring diabetes mellitus in childhood, adolescence, and early adulthood: a nationwide population-based cohort study
Source: BMC Med. 2023 Feb 16;21:59. doi: 10.1186/s12916-023-02762-5 (PMC9933265; doi:10.1186/s12916-023-02762-5)
Supplement: Supplementary file 1 — Additional file 1: Table S1. The information of registers used in this study. Table S2. Detailed information of methods used to identify maternal hypertensive disorders during pregnancy. Table S3. Detailed information of methods used to identify offspring diabetes. Table S4. Associations between maternal HDP and diabetes in offspring by baseline characteristics. Table S5. Sensitivity analyses of the association between maternal HDP and diabetes in offspring. Table S6. Association between maternal HDP and diabetes in offspring born after 1994. Table S7. Associations between maternal HDP and diabetes in offspring with additional adjustment for low birth weight. Table S8. Association between maternal HDP and diabetes in offspring born before 2014. Table S9. Associations between maternal HDP and diabetes in offspring after excluding those diabetic patients identified through age. [file 12916_2023_2762_MOESM1_ESM.doc]

**Table S1.** The information of registers used in this study ^a^

| No. | Registry | Year of established | Information |
| --- | --- | --- | --- |
| 1 | The Danish Civil Registration System | 1968– | The Danish Civil Registration System includes the unique 10-digit individual personal identification number, date and place of birth, sex, current address, parents, marital status, spouse, children, and other vital statistics. |
| 2 | The Danish Medical Birth Registry | 1968– | The Danish Medical Birth Registry includes individual personal identification number of newborns, mothers, and fathers, birth characteristics such as sex, date of birth, gestational age, birth weight, 5-min Apgar score, parity, singleton or not, and maternal characteristics such as maternal smoking during pregnancy, maternal pre-pregnancy body mass index. |
| 3 | The Danish National Patient Register | 1977– | The Danish National Patient Register contains data on inpatient from 1977, and data on outpatient and emergency are included from 1995 and onwards. Using the Danish National Patient Registry covering all Danish hospitals, we identified the study population, date of contact, comorbidities within the study population, date and procedures of surgery, and non-fatal endpoints. Each hospital discharge or outpatient visit is recorded with one primary and potentially several secondary discharge diagnoses in the registry, each classified according to the appropriate International Classification of Disease codes (ICD, ICD-8 codes during 1977–1993 and ICD-10 codes from 1994). |
| 4 | The Danish National Prescription Registry | 1995– | The Danish National Prescription Registry records information on all prescriptions dispensed in Danish community pharmacies since 1995, including dispensing date and drug class according to the Anatomical Therapeutic Chemical classification system. |
| 5 | The Danish National Diabetes Register | 1977– | The Danish National Diabetes Register was established based on data from existing Danish health registers, including from the Danish National Patient Register, the Danish National Prescription Registry, the National Health Insurance Service Register, and the Civil Registration System. The Danish National Diabetes Register contains data on individuals diabetes diagnosis by general and specialist practitioners in Denmark. |
| 6 | The Danish Integrated Database for Longitudinal Labour Market Research | 1981– | The Danish Integrated Database for Longitudinal Labour Market Research contains information on personal labour market affiliation and socioeconomic data such as education. |

**^a^**Denmark has been providing universal tax-funded public welfare, including the provision of universal health services, education, student aid, unemployment insurance, and disability pensions. Of these, healthcare guarantee unrestricted access to general practitioners and hospitals, as well as partial reimbursement of prescription drug treatment. Since the 1960s, all live births and new residents in Denmark have been assigned a unique individual personal identification number (Central Personal Register number, CPR). Different National Registers are linkages by the CPR number, which is encrypted by a unique code and ensures that national data protection, privacy, and confidentiality requirements are fulfilled

**Table S2. Detailed information of methods used to identify maternal hypertensive disorders during pregnancy**

| Hypertensive disorders during pregnancy | ICD-8 (1978–1993) | ICD-10 (1994–2018) |
| --- | --- | --- |
| Chronic hypertension | 40009, 40019, 40029, 40039, 40099, 40199 | I10, I11, I12, I13, I15, O10, O11 |
| Gestational hypertension | 63700, 76029 | O13, O16 |
| Pre-eclampsia ^*^ | 63703, 63704, 63709, 63719 | O14.0, O14.1, O14.2, O14.9, O15 |
| Moderate pre-eclampsia | 63703 | O14.0 |
| Severe pre-eclampsia | 63704 | O14.1 |
| HELLP syndrome |  | O14.2 |
| Unspecified pre-eclampsia | 63709 | O14.9 |
| Eclampsia | 63719 | O15 |

ICD: International Statistical Classification of Diseases; ^*^pre-eclampsia includes eclampsia and the HELLP syndrome.

**Table S3. Detailed information of methods used to identify offspring diabetes**

| Priority | Registry | Offspring diabetes | | | | |
| --- | --- | --- | --- | --- | --- | --- |
|  |  | Type 1 diabetes |  | Type 2 diabetes |  | Gestational diabetes |
| 1 | Danish National Diabetes Register; the Danish National Patient Registry (1987–2018) | ICD-8: 249; ICD-10: E10, O24.0 |  | ICD-8: 250; ICD-10: E11, O24.1 |  | ICD-8: 634.74, Y6449; ICD-10: O24.4, O24.9 |
| 2 | The Danish National Prescription Registry (1994–2016) | More than one redeemed prescriptions for insulin (ATC code: A10A) |  | Receipt of chiropody for diabetic patients or more than one redeemed prescriptions for oral anti-diabetics (ATC code: A10B) |  |  |
| 3 | The Danish National Diabetes Register (1978–1986) | The age of onset of diabetes <30 years |  | The age of onset of diabetes ≥30 years |  |  |

ICD: International Statistical Classification of Diseases; ATC code: Anatomical Therapeutic Chemical classification codes

**Table S4.** **Associations between maternal HDP and diabetes in offspring by baseline characteristics**

| **Characteristics** | **Maternal HDP** | | |  | **Hypertension** | | |  | **Preeclampsia or eclampsia** | | |
| --- | --- | --- | --- | --- | --- | --- | --- | --- | --- | --- | --- |
|  | **No. of DM cases** | **Rate (1/1000 person-years)** | **HR (95% CI)** |  | **No. of DM cases** | **Rate (1/1000 person-years)** | **HR (95% CI)** |  | **No. of DM cases** | **Rate (1/1000 person-years)** | **HR (95% CI)** |
| **Singleton** |  |  |  |  |  |  |  |  |  |  |  |
| No | 65 | 0.53 | 1.34 (1.03–1.74) |  | 11 | 0.55 | 1.54 (0.85–2.80) |  | 54 | 0.53 | 1.31 (0.98–1.74) |
| Yes | 1182 | 0.69 | 1.27 (1.19–1.34) |  | 317 | 0.66 | 1.32 (1.18–1.48) |  | 865 | 0.70 | 1.25 (1.16–1.33) |
| **Sex** |  |  |  |  |  |  |  |  |  |  |  |
| Male | 388 | 0.41 | 1.17 (1.06–1.30) |  | 114 | 0.44 | 1.29 (1.07–1.56) |  | 274 | 0.39 | 1.13 (1.00–1.27) |
| Female | 859 | 0.98 | 1.32 (1.23–1.41) |  | 214 | 0.89 | 1.35 (1.18–1.54) |  | 645 | 1.01 | 1.31 (1.21–1.41) |
| **Calendar year of birth** |  |  |  |  |  |  |  |  |  |  |  |
| 1978–1987 | 704 | 1.07 | 1.35 (1.25–1.46) |  | 176 | 1.19 | 1.50 (1.29–1.74) |  | 528 | 1.03 | 1.30 (1.20–1.42) |
| 1988–1997 | 351 | 0.60 | 1.21 (1.09–1.35) |  | 76 | 0.59 | 1.19 (0.95–1.49) |  | 275 | 0.60 | 1.22 (1.08–1.37) |
| 1998–2007 | 140 | 0.35 | 1.07 (0.90–1.27) |  | 54 | 0.39 | 1.15 (0.88–1.51) |  | 86 | 0.33 | 1.03 (0.83–1.28) |
| 2008–2018 | 52 | 0.28 | 1.18 (0.89–1.57) |  | 22 | 0.27 | 1.13 (0.73–1.73) |  | 30 | 0.29 | 1.22 (0.85–1.77) |
| **Maternal country of origin** |  |  |  |  |  |  |  |  |  |  |  |
| Others | 62 | 0.65 | 1.54 (1.19–1.99) |  | 13 | 0.53 | 1.45 (0.84–2.51) |  | 49 | 0.70 | 1.57 (1.18–2.08) |
| Danish origin | 1183 | 0.68 | 1.25 (1.18–1.33) |  | 315 | 0.66 | 1.32 (1.18–1.48) |  | 868 | 0.69 | 1.23 (1.15–1.32) |
| **Parity** |  |  |  |  |  |  |  |  |  |  |  |
| 1 | 699 | 0.64 | 1.21 (1.12–1.31) |  | 147 | 0.62 | 1.27 (1.08–1.49) |  | 552 | 0.64 | 1.19 (1.10–1.30) |
| 2 | 339 | 0.70 | 1.33 (1.20–1.49) |  | 113 | 0.68 | 1.43 (1.19–1.73) |  | 226 | 0.71 | 1.29 (1.13–1.47) |
| ≥3 | 209 | 0.82 | 1.38 (1.20–1.59) |  | 68 | 0.71 | 1.31 (1.03–1.67) |  | 141 | 0.89 | 1.42 (1.20–1.68) |
| **Maternal age at delivery** |  |  |  |  |  |  |  |  |  |  |  |
| <20 | 64 | 1.10 | 1.27 (0.99–1.64) |  | 8 | 1.17 | 1.30 (0.65–2.62) |  | 56 | 1.09 | 1.27 (0.97–1.66) |
| 20–24 | 351 | 0.87 | 1.22 (1.10–1.36) |  | 65 | 0.89 | 1.21 (0.94–1.54) |  | 286 | 0.87 | 1.23 (1.09–1.38) |
| 25–29 | 418 | 0.65 | 1.27 (1.15–1.40) |  | 108 | 0.67 | 1.35 (1.11–1.63) |  | 310 | 0.64 | 1.24 (1.11–1.39) |
| 30–34 | 276 | 0.59 | 1.33 (1.17–1.50) |  | 93 | 0.60 | 1.41 (1.15–1.74) |  | 183 | 0.59 | 1.29 (1.11–1.49) |
| ≥35 | 138 | 0.53 | 1.27 (1.07–1.51) |  | 54 | 0.52 | 1.33 (1.01–1.75) |  | 84 | 0.53 | 1.23 (0.99–1.54) |
| **Maternal BMI before childbirth** |  |  |  |  |  |  |  |  |  |  |  |
| <18.5 | – | – | – |  | – | – | – |  | – | – | – |
| 18.5–24.9 | 35 | 0.24 | 0.93 (0.66–1.30) |  | 15 | 0.24 | 0.94 (0.56–1.56) |  | 20 | 0.23 | 0.92 (0.59–1.44) |
| 25.0–29.9 | 36 | 0.45 | 1.70 (1.20–2.41) |  | 18 | 0.54 | 1.94 (1.20–3.12) |  | 18 | 0.39 | 1.52 (0.94–2.44) |
| ≥30.0 | 27 | 0.34 | 1.17 (0.78–1.76) |  | 11 | 0.31 | 1.05 (0.57–1.94) |  | 16 | 0.37 | 1.28 (0.76–2.13) |
| Unknown | 1146 | 0.75 | 1.27 (1.20–1.35) |  | 283 | 0.77 | 1.35 (1.20–1.51) |  | 863 | 0.75 | 1.25 (1.17–1.34) |
| **Maternal smoking during pregnancy**^a^ |  |  |  |  |  |  |  |  |  |  |  |
| No | 305 | 0.39 | 1.19 (1.05–1.33) |  | 97 | 0.38 | 1.19 (0.97–1.46) |  | 208 | 0.40 | 1.18 (1.03–1.36) |
| Yes | 67 | 0.43 | 1.11 (0.87–1.42) |  | 17 | 0.37 | 1.00 (0.62–1.61) |  | 50 | 0.45 | 1.15 (0.87–1.53) |
| Unknown | 875 | 0.97 | 1.31 (1.22–1.40) |  | 214 | 1.07 | 1.43 (1.25–1.64) |  | 661 | 0.95 | 1.28 (1.18–1.38) |
| **Maternal educational level** |  |  |  |  |  |  |  |  |  |  |  |
| 0–9 | 597 | 1.00 | 1.33 (1.22–1.44) |  | 143 | 1.05 | 1.44 (1.22–1.70) |  | 454 | 0.98 | 1.30 (1.18–1.42) |
| 10–14 | 461 | 0.57 | 1.20 (1.09–1.32) |  | 129 | 0.58 | 1.29 (1.08–1.53) |  | 332 | 0.57 | 1.17 (1.05–1.30) |
| ≥15 | 177 | 0.43 | 1.24 (1.07–1.44) |  | 55 | 0.40 | 1.21 (0.92–1.58) |  | 122 | 0.45 | 1.26 (1.05–1.50) |
| Unknown | 12 | 0.87 | 1.41 (0.78–2.53) |  | – | – | – |  | 11 | 1.09 | 1.80 (0.98–3.31) |
| **Maternal cohabitation at birth** |  |  |  |  |  |  |  |  |  |  |  |
| No | 579 | 0.67 | 1.27 (1.17–1.38) |  | 126 | 0.59 | 1.23 (1.03–1.47) |  | 453 | 0.70 | 1.28 (1.17–1.41) |
| Yes | 668 | 0.69 | 1.27 (1.17–1.37) |  | 202 | 0.71 | 1.40 (1.22–1.61) |  | 466 | 0.68 | 1.21 (1.11–1.33) |
| **Maternal residence at birth** |  |  |  |  |  |  |  |  |  |  |  |
| Copenhagen | 94 | 0.59 | 1.41 (1.14–1.73) |  | 12 | 0.31 | 0.91 (0.51–1.61) |  | 82 | 0.68 | 1.53 (1.22–1.91) |
| Big cities ≥100,000 inhabitants | 179 | 0.74 | 1.37 (1.18–1.60) |  | 73 | 0.86 | 1.54 (1.22–1.94) |  | 106 | 0.68 | 1.28 (1.05–1.55) |
| Other | 974 | 0.68 | 1.24 (1.16–1.32) |  | 243 | 0.64 | 1.30 (1.15–1.48) |  | 731 | 0.69 | 1.22 (1.13–1.31) |
| **Maternal income at birth** |  |  |  |  |  |  |  |  |  |  |  |
| Less than lowest tertiles | 247 | 0.91 | 1.51 (1.33–1.72) |  | 63 | 0.86 | 1.50 (1.17–1.92) |  | 184 | 0.92 | 1.52 (1.31–1.76) |
| lowest and middle tertiles | 311 | 0.65 | 1.11 (0.99–1.25) |  | 66 | 0.56 | 1.01 (0.79–1.28) |  | 245 | 0.68 | 1.14 (1.01–1.30) |
| Middle and highest tertiles | 316 | 0.64 | 1.26 (1.12–1.41) |  | 91 | 0.63 | 1.37 (1.11–1.68) |  | 225 | 0.64 | 1.22 (1.07–1.39) |
| More than highest tertiles | 255 | 0.54 | 1.30 (1.14–1.47) |  | 71 | 0.51 | 1.34 (1.06–1.70) |  | 184 | 0.55 | 1.28 (1.10–1.48) |
| Unknown | 118 | 1.03 | 1.26 (1.05–1.52) |  | 37 | 1.40 | 1.79 (1.29–2.48) |  | 81 | 0.92 | 1.11 (0.89–1.39) |
| **Maternal diabetes history before childbirth** |  |  |  |  |  |  |  |  |  |  |  |
| No | 1137 | 0.64 | 1.27 (1.19–1.34) |  | 288 | 0.60 | 1.32 (1.17–1.48) |  | 849 | 0.66 | 1.25 (1.16–1.34) |
| Yes | 110 | 1.79 | 1.25 (1.02–1.54) |  | 40 | 1.72 | 1.46 (1.06–2.02) |  | 70 | 1.83 | 1.16 (0.90–1.49) |
| **Paternal diabetes history before childbirth** |  |  |  |  |  |  |  |  |  |  |  |
| No | 1214 | 0.67 | 1.27 (1.20–1.35) |  | 317 | 0.64 | 1.34 (1.19–1.49) |  | 897 | 0.68 | 1.25 (1.17–1.34) |
| Yes | 29 | 2.74 | 1.17 (0.80–1.71) |  | 11 | 3.22 | 1.42 (0.78–2.59) |  | 18 | 2.51 | 1.06 (0.66–1.70) |

^a^Information on maternal smoking during pregnancy was available from 1991 to 2018

Cox regression models (using offspring's age as time scale) were adjusted for calendar year of birth, maternal age, maternal country of origin, maternal residence at birth, maternal cohabitation at birth, maternal educational level, maternal income categories at birth, maternal pre-pregnancy BMI, maternal smoking status during pregnancy, singleton status, maternal diabetes history before childbirth, paternal diabetes history before childbirth, parity, and sex of offspring

*DM*, diabetes mellitus; *HDP*, hypertensive disorders during pregnancy; *HR*, hazard ratio; *CI*, confidence interval

**Table S5.** **Sensitivity analyses of the association between maternal HDP and diabetes in offspring**

| **Outcomes of offspring** | **Exposure** | **No. of diabetes cases** | **Rate (1/1000 person-years)** | **cHR^a^ (95%CI)** | **aHR^b^ (95%CI)** |
| --- | --- | --- | --- | --- | --- |
| **Additional adjustment for paternal hypertension** | **No maternal HDP** | 23645 | 0.52 | 1.00 (Reference) | 1.00 (Reference) |
|  | **Maternal HDP** | 1247 | 0.68 | 1.33 (1.26–1.41) | 1.27 (1.20–1.34) |
|  | **Preeclampsia or eclampsia** | 919 | 0.69 | 1.30 (1.22–1.39) | 1.25 (1.17–1.33) |
|  | **Preeclampsia** | 915 | 0.69 | 1.31 (1.22–1.40) | 1.25 (1.17–1.34) |
|  | Moderate | 713 | 0.71 | 1.29 (1.20–1.39) | 1.25 (1.16–1.34) |
|  | Severe | 135 | 0.65 | 1.38 (1.16–1.63) | 1.28 (1.08–1.51) |
|  | Unspecified | 63 | 0.75 | 1.41 (1.10–1.80) | 1.34 (1.05–1.72) |
|  | **Hypertension** | 328 | 0.66 | 1.43 (1.29–1.60) | 1.33 (1.19–1.48) |
|  | Chronic | 90 | 0.50 | 1.44 (1.17–1.78) | 1.22 (0.99–1.51) |
|  | Gestational | 238 | 0.75 | 1.43 (1.26–1.63) | 1.37 (1.21–1.56) |
|  |  |  |  |  |  |
| **Offspring born after 1991 (additional adjustment for maternal smoking during pregnancy)** | **No maternal HDP** | 7138 | 0.32 | 1.00 (Reference) | 1.00 (Reference) |
|  | **Maternal HDP** | 358 | 0.39 | 1.25 (1.12–1.39) | 1.17 (1.05–1.30) |
|  | **Preeclampsia or eclampsia** | 243 | 0.39 | 1.22 (1.07–1.39) | 1.16 (1.02–1.32) |
|  | **Preeclampsia** | 243 | 0.39 | 1.23 (1.08–1.40) | 1.17 (1.03–1.33) |
|  | Moderate | 171 | 0.39 | 1.20 (1.03–1.40) | 1.14 (0.98–1.33) |
|  | Severe | 39 | 0.33 | 1.05 (0.76–1.43) | 0.99 (0.72–1.36) |
|  | Unspecified | 29 | 0.79 | 2.34 (1.63–3.37) | 2.29 (1.59–3.31) |
|  | **Hypertension** | 115 | 0.38 | 1.31 (1.09–1.57) | 1.19 (0.99–1.43) |
|  | Chronic | 58 | 0.40 | 1.41 (1.09–1.83) | 1.23 (0.95–1.60) |
|  | Gestational | 57 | 0.37 | 1.22 (0.94–1.58) | 1.14 (0.88–1.49) |
|  |  |  |  |  |  |
| **After excluding mothers diagnosed with multiple types of hypertension in one pregnancy** | **No maternal HDP** | 23,833 | 0.52 | 1.00 (Reference) | 1.00 (Reference) |
|  | **Maternal HDP** | 1059 | 0.68 | 1.31 (1.23–1.39) | 1.25 (1.17–1.33) |
|  | **Preeclampsia or eclampsia** | 755 | 0.70 | 1.27 (1.18–1.37) | 1.22 (1.14–1.32) |
|  | **Preeclampsia** | 755 | 0.70 | 1.28 (1.19–1.37) | 1.23 (1.14–1.32) |
|  | Moderate | 613 | 0.70 | 1.25 (1.16–1.36) | 1.21 (1.12–1.32) |
|  | Severe | 82 | 0.66 | 1.38 (1.11–1.72) | 1.27 (1.02–1.58) |
|  | Unspecified | 57 | 0.78 | 1.42 (1.09–1.84) | 1.36 (1.05–1.76) |
|  | **Hypertension** | 304 | 0.65 | 1.40 (1.25–1.57) | 1.31 (1.17–1.47) |
|  | Chronic | 66 | 0.45 | 1.32 (1.04–1.69) | 1.12 (0.88–1.43) |
|  | Gestational | 238 | 0.75 | 1.42 (1.25–1.62) | 1.37 (1.21–1.56) |
|  |  |  |  |  |  |
| **Additional adjustment for maternal obesity before childbirth** | **No maternal HDP** | 23,645 | 0.52 | 1.00 (Reference) | 1.00 (Reference) |
|  | **Maternal HDP** | 1247 | 0.68 | 1.33 (1.26–1.41) | 1.25 (1.18–1.32) |
|  | **Preeclampsia or eclampsia** | 919 | 0.69 | 1.30 (1.22–1.39) | 1.23 (1.15–1.32) |
|  | **Preeclampsia** | 915 | 0.69 | 1.31 (1.22–1.40) | 1.24 (1.16–1.32) |
|  | Moderate | 713 | 0.71 | 1.29 (1.20–1.39) | 1.23 (1.14–1.33) |
|  | Severe | 135 | 0.65 | 1.38 (1.16–1.63) | 1.27 (1.07–1.50) |
|  | Unspecified | 63 | 0.75 | 1.41 (1.10–1.80) | 1.33 (1.04–1.71) |
|  | **Hypertension** | 328 | 0.66 | 1.43 (1.29–1.60) | 1.30 (1.17–1.45) |
|  | Chronic | 90 | 0.50 | 1.44 (1.17–1.78) | 1.20 (0.97–1.47) |
|  | Gestational | 238 | 0.75 | 1.43 (1.26–1.63) | 1.35 (1.19–1.53) |

Cox regression models (using offspring's age as time scale) were adjusted for calendar year of birth, maternal age, maternal country of origin, maternal residence at birth, maternal cohabitation at birth, maternal educational level, maternal income categories at birth, maternal pre-pregnancy BMI, maternal smoking status during pregnancy, singleton status, maternal diabetes history before childbirth, paternal diabetes history before childbirth, parity, and sex of offspring

As data on smoking status during pregnancy was available only from 1991, an analysis with additional adjustment for smoking status during pregnancy was restricted to offspring born after 1991

*aHR*, adjusted hazard ratio; *cHR*, crude hazard ratio; *HDP*, hypertensive disorders during pregnancy; *HELLP*, hemolysis, elevated liver enzymes, and low platelet; *CI*, confidence interval

**Table S6. Association between maternal HDP and diabetes in offspring born after 1994**

| **Outcomes of offspring** | **Exposure** | **No. of diabetes cases** | **Rate (1/1000 person–years)** | **cHR^a^ (95%CI)** | **aHR^b^ (95%CI)** |
| --- | --- | --- | --- | --- | --- |
| **Type 1 diabetes** | **No maternal HDP** | 4646 | 0.27 | 1.00 (Reference) | 1.00 (Reference) |
|  | **Maternal HDP** | 227 | 0.31 | 1.17 (1.02–1.33) | 1.07 (0.94–1.23) |
|  |  |  |  |  |  |
| **Type 2 diabetes** | **No maternal HDP** | 267 | 0.02 | 1.00 (Reference) | 1.00 (Reference) |
|  | **Maternal HDP** | 20 | 0.03 | 1.91 (1.21–3.01) | 1.90 (1.19–3.02) |
|  |  |  |  |  |  |
| **Gestational diabetes^*^** | **No maternal HDP** | 149 | 1.17 | 1.00 (Reference) | 1.00 (Reference) |
|  | **Maternal HDP** | 11 | 2.58 | 2.25 (1.22–4.14) | 2.24 (1.20–4.20) |

^*^The analysis of gestational diabetes was restricted to female offspring who had been pregnant

^a^Model 1: using offspring's age as time scale

^b^Model 2: using offspring's age as time scale; adjusted for calendar year of birth, maternal age at birth, maternal country of origin, maternal residence at birth, maternal cohabitation at birth, maternal educational level, maternal income categories at birth, maternal pre-pregnancy BMI, maternal smoking status during pregnancy, singleton status, maternal diabetes history before childbirth, paternal diabetes history before childbirth, parity, and sex of offspring

*aHR*, adjusted hazard ratio; *cHR*, crude hazard ratio; *HDP*, hypertensive disorders during pregnancy; *CI*, confidence interval

**Table S7. Associations between maternal HDP and diabetes in offspring with additional adjustment for low birth weight**

| **Outcomes of offspring** | **Exposure** | **No. of diabetes cases** | **Rate (1/1000 person-years)** | **Total effect^a^ (95%CI)** | **Directed effect^b^ (95%CI)** |
| --- | --- | --- | --- | --- | --- |
| **Diabetes mellitus** | **No maternal HDP** | 23,645 | 0.52 | 1.00 (Reference) | 1.00 (Reference) |
|  | **Maternal HDP** | 1247 | 0.68 | 1.27 (1.20–1.34) | 1.23 (1.16–1.30) |
|  |  |  |  |  |  |
| **Type 1 diabetes** | **No maternal HDP** | 10,599 | 0.23 | 1.00 (Reference) | 1.00 (Reference) |
|  | **Maternal HDP** | 493 | 0.27 | 1.08 (0.98–1.18) | 1.07 (0.97–1.17) |
|  |  |  |  |  |  |
| **Type 2 diabetes** | **No maternal HDP** | 3872 | 0.09 | 1.00 (Reference) | 1.00 (Reference) |
|  | **Maternal HDP** | 252 | 0.14 | 1.57 (1.38–1.78) | 1.49 (1.31–1.70) |
|  |  |  |  |  |  |
| **Gestational diabetes^*^** | **No maternal HDP** | 9174 | 1.14 | 1.00 (Reference) | 1.00 (Reference) |
|  | **Maternal HDP** | 502 | 1.62 | 1.37 (1.25–1.49) | 1.31 (1.20–1.44) |

**^*^**The analysis of gestational diabetes was restricted to female offspring who had been pregnant

^a^Total effect: using offspring's age as time scale; adjusted for calendar year of birth, maternal age at birth, maternal country of origin, maternal residence at birth, maternal cohabitation at birth, maternal educational level, maternal income categories at birth, maternal pre-pregnancy BMI, maternal smoking status during pregnancy, singleton status, maternal diabetes history before childbirth, paternal diabetes history before childbirth, parity, and sex of offspring

^b^Directed effect: using offspring's age as time scale; additionally adjusted for low birth weight in addition to the above factors in total effect

*aHR*, adjusted hazard ratio; *cHR*, crude hazard ratio; *HDP*, hypertensive disorders during pregnancy; *CI*, confidence interval

**Table S8. Association between maternal HDP and diabetes in offspring born before 2014**

| **Exposure** | **No. of diabetes cases** | **Rate (1/1000 person-years)** | **cHR^a^ (95%CI)** | **aHR^b^ (95%CI)** |
| --- | --- | --- | --- | --- |
| **No maternal HDP** | 23,566 | 0.53 | 1.00 (Reference) | 1.00 (Reference) |
| **Maternal HDP** | 1241 | 0.69 | 1.33 (1.26–1.41) | 1.27 (1.20–1.34) |
| **Preeclampsia or eclampsia** | 915 | 0.70 | 1.30 (1.22–1.39) | 1.25 (1.17–1.33) |
| **Preeclampsia** | 911 | 0.70 | 1.31 (1.22–1.39) | 1.25 (1.17–1.34) |
| Moderate | 711 | 0.71 | 1.29 (1.20–1.39) | 1.25 (1.16–1.34) |
| Severe | 134 | 0.66 | 1.37 (1.16–1.63) | 1.27 (1.07–1.51) |
| HELLP syndrome^*^ | – | – | – | – |
| Unspecified | 63 | 0.76 | 1.41 (1.10–1.80) | 1.35 (1.05–1.72) |
| **Eclampsia**^*^ | – | – | – | – |
| **Hypertension** | 326 | 0.67 | 1.44 (1.29–1.60) | 1.33 (1.20–1.49) |
| Chronic | 89 | 0.51 | 1.45 (1.18–1.79) | 1.23 (1.00–1.52) |
| Gestational | 237 | 0.76 | 1.43 (1.26–1.63) | 1.38 (1.21–1.57) |

**^*^**Number of cases for HELLP syndrome and eclampsia are less than 6 and not allowed to report due to privacy protection, therefore, we did not report results of HELLP syndrome and eclampsia

^a^Total effect: using offspring's age as time scale; adjusted for calendar year of birth, maternal age at birth, maternal country of origin, maternal residence at birth, maternal cohabitation at birth, maternal educational level, maternal income categories at birth, maternal pre-pregnancy BMI, maternal smoking status during pregnancy, singleton status, maternal diabetes history before childbirth, paternal diabetes history before childbirth, parity, and sex of offspring

^b^Directed effect: using offspring's age as time scale; additionally adjusted for low birth weight in addition to the above factors in total effect

*aHR*, adjusted hazard ratio; *cHR*, crude hazard ratio; *HDP*, hypertensive disorders during pregnancy; *CI*, confidence interval

**Table S9. Associations between maternal HDP and diabetes in offspring after excluding those diabetic patients identified through age**

| **Exposure** | **No. of diabetes cases** | **Rate (1/1000 person-years)** | **cHR^a^ (95%CI)** | **aHR^b^ (95%CI)** |
| --- | --- | --- | --- | --- |
| **No maternal HDP** | 23,454 | 0.52 | 1.00 (Reference) | 1.00 (Reference) |
| **Maternal HDP** | 1240 | 0.68 | 1.34 (1.26–1.42) | 1.27 (1.20–1.35) |
| **Preeclampsia or eclampsia** | 913 | 0.68 | 1.30 (1.22–1.39) | 1.25 (1.17–1.34) |
| **Preeclampsia** | 910 | 0.69 | 1.31 (1.23–1.40) | 1.26 (1.18–1.34) |
| Moderate | 709 | 0.70 | 1.29 (1.20–1.39) | 1.25 (1.16–1.35) |
| Severe | 134 | 0.64 | 1.38 (1.16–1.63) | 1.28 (1.08–1.51) |
| HELLP syndrome^*^ | – | – | – | – |
| Unspecified | 63 | 0.75 | 1.41 (1.10–1.81) | 1.35 (1.06–1.73) |
| **Eclampsia**^*^ | – | – | – | – |
| **Hypertension** | 327 | 0.65 | 1.45 (1.30–1.61) | 1.34 (1.20–1.49) |
| Chronic | 90 | 0.50 | 1.47 (1.19–1.80) | 1.23 (1.00–1.51) |
| Gestational | 237 | 0.74 | 1.43 (1.26–1.63) | 1.38 (1.21–1.57) |

**^*^**Number of cases for HELLP syndrome and eclampsia are less than 6 and not allowed to report due to privacy protection, therefore, we did not report results of HELLP syndrome and eclampsia

^a^Model 1: using offspring's age as time scale

^b^Model 2: using offspring's age as time scale; adjusted for calendar year of birth, maternal age at birth, maternal country of origin, maternal residence at birth, maternal cohabitation at birth, maternal educational level, maternal income categories at birth, maternal pre-pregnancy BMI, maternal smoking status during pregnancy, singleton status, maternal diabetes history before childbirth, paternal diabetes history before childbirth, parity, and sex of offspring

*aHR*, adjusted hazard ratio; *cHR*, crude hazard ratio; *HDP*, hypertensive disorders during pregnancy; *HELLP*, hemolysis, elevated liver enzymes, and low platelet; *CI*, confidence interval
